# Metabolomics Reveals 5-Aminolevulinic Acid Improved the Ability of Tea Leaves (*Camellia sinensis* L.) against Cold Stress

**DOI:** 10.3390/metabo12050392

**Published:** 2022-04-26

**Authors:** Fei Yan, Dong Qu, Xiaohua Chen, Haitao Zeng, Xinsheng Li, Ching Yuan Hu

**Affiliations:** 1 Shaanxi Provincial Bioresource Key Laboratory, College of Biological Science and Engineering, Shaanxi University of Technology, Hanzhong 723000, China; yanfei@snut.edu.cn (F.Y.); chenxiaohua@snut.edu.cn (X.C.); zenghaitao@snut.edu.cn (H.Z.); lixinsheng@snut.edu.cn (X.L.); 2 Qinling-Bashan Mountains Bioresources Comprehensive Development C. I. C, Hanzhong 723001, China; 3 Qinba State Key Laboratory of Biological Resources and Ecological Environment, Hanzhong 723001, China; 4 Department of Human Nutrition, Food and Animal Sciences, College of Tropical Agriculture and Human Resources, University of Hawaii at Manoa, 1955 East-West Road, AgSci. 415J, Honolulu, HI 96822, USA

**Keywords:** secondary metabolites, soluble carbohydrates, abiotic stress

## Abstract

Tea is an important woody crop whose cultivation is severely limited by cold stress. Although 5-aminolevulinic acid (ALA) is known to be effective in alleviating abiotic stresses in plants, knowledge of the detailed metabolic response of tea plants to exogenous ALA-induced cold resistance is still limited—a lack which restricts our ability to protect tea plants from cold stress. In the present study, we performed an in-depth metabolomics analysis to elucidate the metabolic responses of tea plants to cold stress and explore the role of ALA in improving tea plants’ cold-resistance capability. Metabolic profiles showed that cold stress altered various metabolisms in tea plants, especially galactose composition and flavonoid contents. Furthermore, exogenous ALA application altered a series of metabolisms associated with cold stress. Importantly, increases in metabolites, including catechin, 3,4-dihydroxyphenylacetic acid and procyanidin B2, involved in the mechanisms of ALA improved tea plants’ cold resistance. Overall, our study deciphered detailed metabolic responses of tea plants to cold stress and elucidated the mechanisms of ALA in enhancing cold resistance through rebuilding compositions of soluble carbohydrates and flavonoids. Therefore, we have provided a basis for exogenous usage of ALA to protect tea plants from cold stress.

## 1. Introduction

Tea plants (*Camellia sinensis* (L.) O. Kuntze) are rich in polyphenols, theanine, caffeine, and vitamins. Teas are known to have various medicinal properties, such as anticancer and blood pressure-lowering effects. Additionally, tea plants are the best biomaterials for the obtainment of catechins and theanine drugs. Tea is the most consumed nonalcoholic beverage worldwide [1]. Thus, the tea plant is one of the most valuable woody economic crops around the world [2]. Tea plants are widely distributed in tropical and subtropical regions, and their particular environments enable them to be adapted to warm temperatures. With the increasing demand for tea, tea planting area is expanding to low-temperature areas; thus, cold stress is a non-negligible problem that affects tea quality and restricts the tea industry’s development [3]. Additionally, as global climate changes intensify, severe environmental issues, particularly colder temperatures, threaten tea plant growth. Lower temperatures during the growing season always negatively affect the accumulation of metabolites in tea shoots or even damage them, threatening tea productivity and quality [4]. Therefore, it is urgent to improve cold resistance in tea plants.

Cold stress affects cell membrane fluidity, the cytoskeleton, free radical production and enzyme activity [1,5,6]. Facing cold stress, plants can deploy a series of reactions to improve their cold resistance in a process called cold acclimation [7]. Cold acclimation involves physiological and biochemical changes, including the activation of cold-related genes and the regulation of osmotic substance production and the antioxidant enzyme system [8]. Therefore, increasing research has investigated the management strategy for tea plants facing cold stress [9,10,11]. Various cold-resistance genes have been isolated and identified from a wide range of plants, including Arabidopsis, potato and tomato [12].

Cold stress causes various metabolic disorders in tea plants by affecting the activity of multiple enzymes, leading to changes in tissue contents of catechins, vitamins and amino acids [2,7,13]. As the main secondary metabolites in tea, flavonoids perform various functions in plant resistance to biotic and especially abiotic stress [14]. Anthocyanins and flavonoid contents significantly changed in the grape epidermis under cold stress [15]. Flavonoids and anthocyanins improved cold resistance in plants by scavenging superoxide anions and hydroxyl radicals [16]. Meanwhile, flavonoids also enhanced cold resistance in plants by increasing contents of proline and soluble sugars through performing signal functions to activate the biosynthesis of soluble carbohydrate [17,18].

Additionally, increasing intracellular soluble sugar content is vital for plants to improve their cold resistance through increasing the concentration of cell fluid and non-frozen water in cells, leading to decreases in the freezing point of cytoplasm [19,20,21]. Thus, glucose metabolism is closely associated with plant response to cold stress. This process is quite complex and involves a series of changes in enzyme activity, gene expression and signal transduction [22]. Cold stress has been shown to affect photosynthesis in plants, leading to a decrease in starch content and increases in soluble sugar content in vivo [23]. Transmission electron microscopy (TEM) of tea leaves shows that, compared to the increased maltose content in leaves, starch grains in chloroplasts decreased or even disappeared after cold acclimation [24]. Many genes that function in starch metabolism were significantly different in *Arabidopsis thaliana* under cold stress [25]. Meanwhile, the soluble sugar content in cells increased in plant tissues upon cold stress and directly affected plants’ abilities to endure cold resistance [25,26]. Cold-resistant plants have higher soluble sugar contents [19,20]. Sugar could act as a signaling molecule to regulate stress response and the development of plants [27,28]. However, the global metabolic alterations in tea tissues under cold stress are still unclear.

Traditional methods to cope with low temperature stress, such as breeding cold-resistant varieties and strengthening field management, are time-consuming and costly. Growth regulators play essential roles in plant resistance to environmental abiotic stress and thus have a broad application prospect in protecting crops from various abiotic stresses [29]. As a metabolic intermediate in higher plants, 5-aminolevulinic acid (ALA) is a precursor of porphyrin synthesis in plant chlorophyll and could improve plant growth under various stresses [30,31,32]. Furthermore, exogenous ALA alleviated drought-induced damage to many plants and directly enhanced photosynthetic capacities to promote plant growth [33,34,35]. Remarkably, ALA treatment improved fruit storage under low temperatures [36]. Furthermore, exogenous ALA treatment improved various metabolic processes related to plant cold resistance, e.g., increasing anthocyanin and soluble sugar contents in plant tissues, implying that ALA treatment may improve plants’ abilities to handle cold stress [37,38,39]. Thus, it is critical to investigate the detailed effects of ALA treatment on tea plants with respect to cold resistance. This information will contribute to the management of tea cultivation under cold stress.

Our previous study indicated that exogenous ALA decreased the electrolyte leakage rate, MDA (malondialdehyde) and H_2_O_2_ accumulation levels and elevated proline content under cold stress. Moreover, tea leaves under cold stress had a significantly higher maximum photosynthetic capacity (Fv/Fm) after spraying with ALA [40]. Thus, ALA application improved tea leaves’ cold resistance by maintaining membrane systems’ stability and the photosynthetic apparatus. In the present study, we employed in-depth untargeted metabolomics to identify detailed metabolic variations in tea plants following cold challenge and explore the chemical mechanisms of ALA treatment in improving cold resistance in tea plants. Overall, our research expands our understanding of plant metabolic responses to cold stress and elucidates the underlying mechanisms of ALA treatment in improving cold resistance in tea plants, contributing to the usage of ALA in protecting tea from cold stress.

## 2. Results

### 2.1. Cold Stress Induced Dramatic Alterations of Secondary Metabolites in Tea Leaves

**Figure 1:**
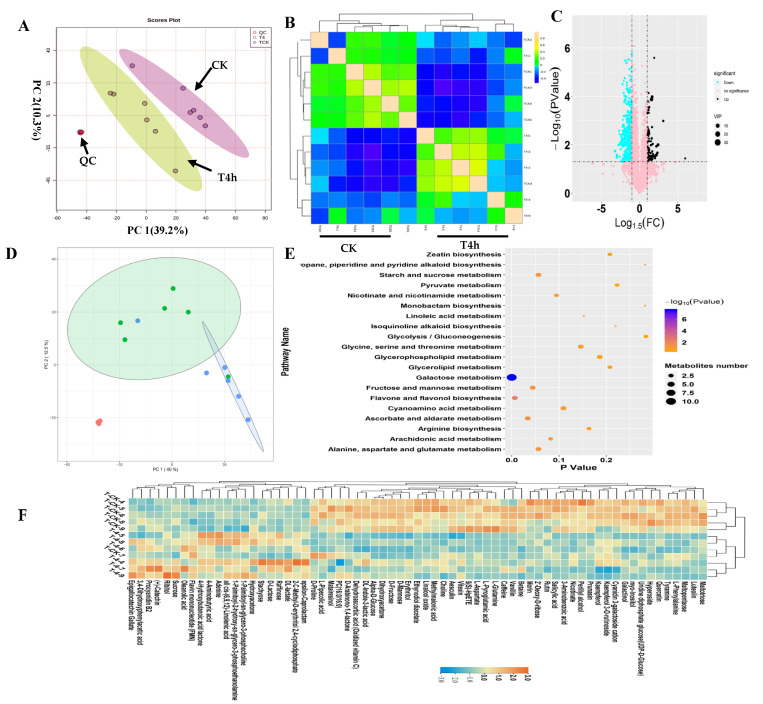
Overview of metabolic patterns of tea following cold stress. (**A**) Results of the PCA analysis on the standardized data matrix. The score plot (left) shows the projection of the samples in the PC1 and PC2 planes. The loading plot shows the distribution of samples from the T4h and control groups, shown in green and blue, respectively. (**B**) Correlation analysis between samples from T4h and control groups. The scale represents the correlation value between two samples. (**C**) Volcano plot showing the differential metabolites in T4h vs. CK (fold changed ≥ 2, *p*-value ≤ 0.05, VIP > 1.0). The vertical coordinate represents the *p*-value of the Student’s *t*-test (−log _10_ *p*-value). The increased and decreased metabolites are shown in black and blue, respectively. The metabolites labeled pink are not significant. The plot size represents the VIP value of the PLSDA model. (**D**) SOM PCA plot distribution exhibiting two metabolic patterns among all samples. The ellipse represents the 95% confidence interval. (**E**) Scatter plot of the most enriched Kyoto Encyclopedia of Genes and Genomes (KEGG) pathways of differential metabolites. (**F**) Heatmap showing the relative levels of differential metabolites with VIP values > 1.0. The upregulated and downregulated metabolites are shown in red and blue, respectively. The scale represents the normalized peak areas of metabolites.

In this study, in-depth untargeted metabolomics was performed to investigate tea leaves’ metabolic alterations before (CK) and after −4 °C treatment (T4h). (Figure 1). To observe the relationship between the metabolic variations and cold treatment, a principal component analysis (PCA) of the metabolic profiles was performed to gain an overview of the metabolic patterns (Figure 1A). The results in two-dimensional space showed that the samples observably formed two regions according to their treatments. One variety stood out: T4h was separated from CK on the first PC, which explained 39.2% variations. Therefore, compared with CK’s metabolites, the cold treatment caused dramatic variations in tea leaves (Figure 1A). Consistently, OPLSDA plots effectively separated the samples into two groups, CK and T4h, according to their treatments (Figure S3). Further unsupervised correlation analysis of metabolic profiles from the T4h and CK groups proved the observable influences of cold treatment on the metabolic patterns of tea leaves. It showed that all samples from T4h clustered together and separated with CK samples clearly (Figure 1B). Moreover, the close clustering of quality control (QC) samples proved our results’ indication that there was no other technological factor causing metabolic variations (Figure 1A,B). Then, we set *p* < 0.05 and |log_2_FC| > 1.0 as a threshold to identify the differential metabolites (Figure 1C). Five-hundred-and-seventy-four metabolites were identified as significantly differential components (Figure 1C). Among all differential metabolites, 474 compounds, such as L-glutamine, 5(S)-HpETE, L-pyroglutamic acid, vanillin and L-aspartate, were downregulated. At the same time, 100 metabolites, such as catechin, 4-hydroxybutanoic acid lactone, 4-aminobutyric acid, oleanolic acid, ribitol and 2-C-methyl-D-erythritol 2,4-cyclodiphosphate, were upregulated (Figure 1C; Table S1). Further, we performed a supervised approach PLSDA to focus specifically on relevant differences among classes and obtained the VIP of metabolites. Thus, we mainly deployed VIP values > 1.0 to calculate the contribution of all metabolites in the cold-induced metabolic variations. The results showed 64 metabolites with VIP values > 1.0. Among them, stachyose, rutin, D-lactose, PC (16:0/16:0), hyperoside, caffeine, quercetin, alpha-d-glucose, raffinose, oxidized vitamin C and epigallocatechin gallate had VIP values > 4.0 (Table S1). These 64 metabolites were the focus of further analysis.

A self-organizing map (SOM) organizes features using intensity trends; thus, samples with similar metabolic constituents are located in an adjacent regions in the SOM coordinate system. The SOM analysis results for all metabolic profiles show that T4h and CK samples were separated into two regions, indicating that the metabolic compositions of tea leaves under cold stress were different from the control. Cold stress can have a non-negligible influence on metabolic composition variation in tea leaves (Figure 1D). We linked the screened 64 differential metabolites to metabolic pathways in the KEGG database and explored the potential metabolic pathways of tea leaves in response to cold temperatures (Figure 1E). The results showed that the main metabolic pathways of differential metabolites associated with cold stress included galactose, flavone and flavonol biosynthesis, ascorbate and aldarate, fructose and mannose, starch and sucrose, alanine, aspartate and glutamate, arachidonic acid, nicotinate and nicotinamide, cyanoamino acid and glycine, serine and threonine (Figure 1E). These results suggest that cold stress mainly affected carbohydrate and flavone biosynthesis.

Furthermore, we performed KEGG enrichment analysis on downregulated and upregulated metabolites (Table S1). The results showed that the main downregulated pathways were galactose metabolism, ATP-binding cassette (ABC) transporters, and biosyntheses of phenylpropanoids, flavone, flavonols and alkaloids derived from ornithine, lysine and nicotinic acid. The upregulated metabolites are mainly involved in galactose metabolism, riboflavin metabolism, phosphotransferase system (PTS), taste transduction and oxidative phosphorylation (Table S1). We noted that galactose metabolism in plants was the most significant pathway to upregulate and downregulate metabolites under cold stress (Figure 1F; Table S1). Additionally, cold stress may suppress flavone metabolism and cause damage to plants’ normal physiological processes.

### 2.2. ALA Altered Flavonoid and Carbohydrate Compositions in Tea Leaves

**Figure 2:**
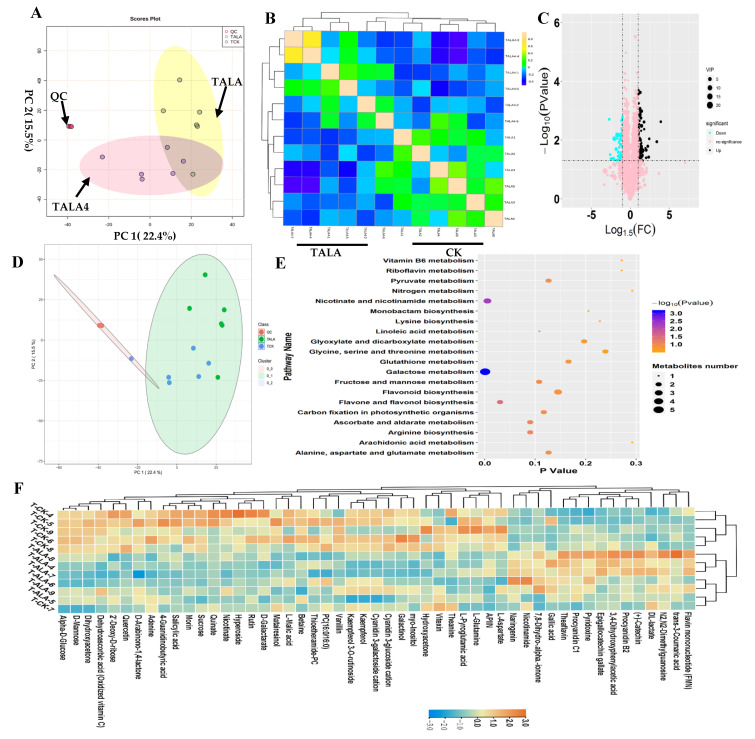
Landscape of metabolic patterns of tea following ALA treatment. (**A**) PCA analysis of metabolic profiles from TALA and control groups. The TALA and control groups’ samples are green and blue in the loading plot, respectively. (**B**) Correlation analysis between samples from TALA and the control. (**C**) Volcano plot showing the differential metabolites in TALA vs. CK comparison. The vertical coordinate represents the *p*-value of the Student’s *t*-test (−log 10 *p*-value). The VIP value of each metabolite from the PLSDA analysis is represented by the plot size. (**D**) SOM PCA plot distribution exhibiting similar metabolic patterns among all samples. The samples with a similar metabolic pattern were labeled by a 95% confidence interval ellipse. (**E**) Scatter plot of the most enriched Kyoto Encyclopedia of Genes and Genomes (KEGG) pathways of differential metabolites with VIP values > 1.0. (**F**) Heatmap showing the relative levels of differential metabolites associated with ALA treatment. The upregulated and downregulated metabolites are shown in red and blue, respectively. The scale represents the normalized peak areas of metabolites.

In this study, we performed a metabolomics analysis based on the LC–MS platform to explore ALA’s influence on metabolic variations in tea leaves (Figure 2). Tea leaves were treated with exogenous ALA (TALA) for two days and then collected for untargeted metabolomics analysis. Tea leaves without exogenous ALA treatment were used as the control (CK). PCA was performed on the metabolic profiles from TALA and CK groups to explore the vast number of multi-dimensional data points generated and discover unbiased relationships (Figure 2A). The results indicated that the two top components representing PC1 and PC2 could explain 37.9% of the variance in the data. From the color-coding of sample points, TALA samples were separated from the control samples, suggesting that ALA treatment effectively altered the metabolic patterns of tea leaves (Figure 2A). The samples were distributed into two groups according to their treatments, TALA and TALA4, using OPLSDA analysis (Figure S3). Then, the unsupervised correlation analysis results showed that samples were clustered together according to their treatments, in agreement with observations from the PCA diagram (Figure 2B). Additionally, the close separation of QC samples supported the credibility of our results (Figure 2A,B). Subsequently, pairwise contrasts within TALA and CK were performed. In total, one-hundred-and-sixty-one metabolites were identified as significantly different metabolites in TALA vs. CK. Among them, sixty-six metabolites were downregulated, including morin, quinate, L-malic acid, vanillin and rutin. Ninety-five metabolites were upregulated, including nicotinamide, theaflavin, procyanidin B2, catechin and FMN (Figure 2C,D; Table S2). Supervised PLSDA calculated the VIP values of each metabolite and evaluated the metabolites’ associations with ALA treatment (Table S2). Ultimately, fifty-one metabolites with VIP values > 1.0 were identified as hub metabolites. These changes included thioetheramide-PC, procyanidin B2, catechin, kaempferol, PC (16:0/16:0), kaempferol 3-O-rutinoside, theanine, morin, dehydroascorbic acid and especially quercetin, and explained most of the ALA-induced metabolite variations (Table S2). Among these fifty-one metabolites, we observed that thirty-five metabolites were decreased and sixteen metabolites increased in tea leaves after ALA treatment (Table S2; Figure 2F). Further SOM analysis showed that ALA treatment can cause dramatic alterations in tea leaves’ metabolic compositions compared to cold stress (Figure 2D). Thus, we propose that ALA promotes cold resistance in tea plants and does not affect the flavor quality of tea.

We performed KEGG pathway enrichment analysis to investigate the effects of ALA treatment on tea leaf metabolites (Figure 2E; Table S2). The results of downregulated metabolites show the overrepresentation of sixty-seven metabolic pathways, including mainly phenylpropanoid, galactose, flavone and flavonol biosynthesis, proximal tubule bicarbonate reclamation, biosynthesis of plant hormones, alkaloids derived from ornithine, lysine and nicotinic acid, the two-component system, flavonoid biosynthesis and ABC transporters (Table S2). Additionally, upregulated metabolites are mainly involved in phenylpropanoid biosynthesis, flavonoid biosynthesis, oxidative phosphorylation, pyruvate, riboflavin, vitamin B6, butirosin and neomycin biosynthesis, phenylalanine metabolism, benzoate degradation via hydroxylation and nicotinate and nicotinamide metabolism (Table S2). We found that ALA mainly affected secondary metabolisms represented by phenylpropanoid and flavonoid biosynthesis (Table S2). Further pathway analysis for all differential metabolites indicated that ALA could affect the pattern of flavonoid biosynthesis involved in plant cold resistance (Figure 2E). In addition, carbohydrates and amino acids are the most significant pathway affected by ALA treatment. Various related pathways are overrepresented among differential metabolites associated with ALA treatment, including galactose, ascorbate and aldarate, arginine biosynthesis, fructose and mannose, linoleic acid, pyruvate and alanine, aspartate and glutamate (Figure 2E).

### 2.3. Exogenous ALA Affected Metabolite Levels Associated with Cold Stress

**Figure 3:**
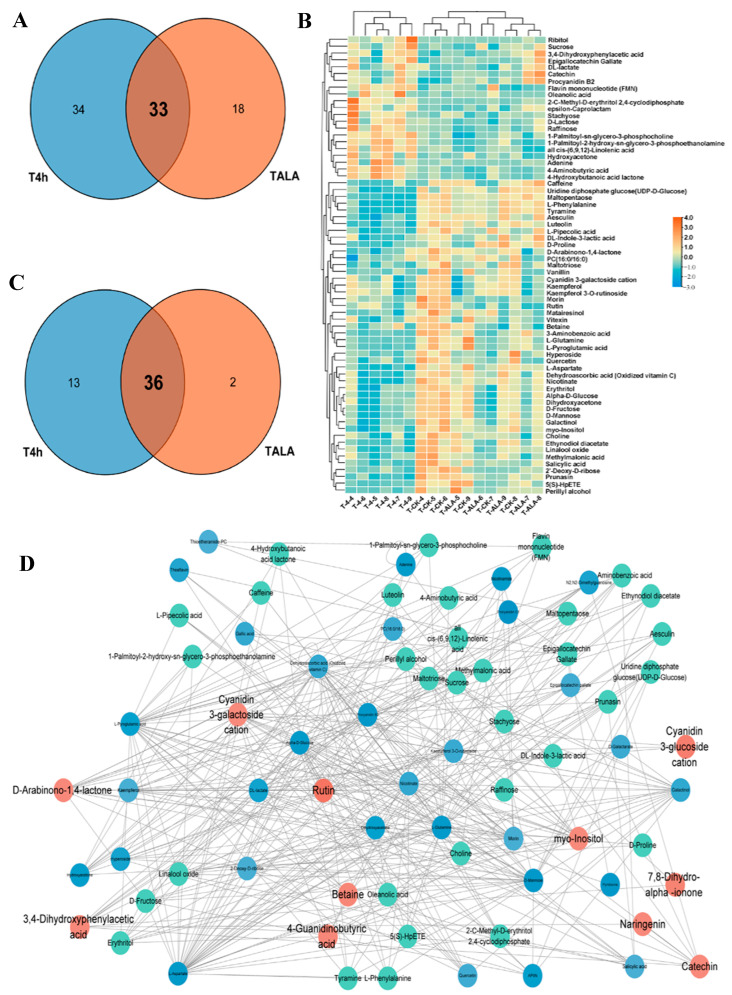
Overlapping of ALA- and cold-induced metabolic alterations. (**A**) Venn diagram of all significantly differential metabolites in T4h vs. CK and TALA vs. CK comparisons. Counts in blue and red denote metabolites from T4h vs. CK and TALA vs. CK, respectively. The shaded area denotes the differential metabolites shared by both comparisons. (**B**) Heatmap displaying the ALA-induced alterations in relative levels of metabolites associated with cold stress. The overlapping area represents the common pathways shared by both comparisons. (**C**) Venn diagram showing the overlapping enriched pathways in the T4h vs. CK and TALA vs. CK comparisons. (**D**) Topological correlation network of metabolites associated with ALA treatment and cold stress.

To analyze the common influences of ALA treatment and cold stress on tea leaves’ metabolic patterns, we compared the differential metabolites identified and metabolic pathways in TALA vs. CK and T4h vs. CK comparisons. The results for metabolites show that these two groups shared a total of thirty-three metabolites (Figure 3A). Furthermore, most of these metabolites were commonly triggered or suppressed in tea leaves after ALA treatments or cold stress alone (Table S3; Figure S1). For example, catechin, 3,4-Dihydroxyphenylacetic acid, procyanidin B2 and FMN in tea leaves were elevated after ALA treatments or cold stress alone (Table S3; Figure S1). Additionally, most flavonoids and carbohydrates, including betaine, cyanidin 3-galactoside cation, D-arabinono-1,4-lactone, kaempferol, kaempferol 3-O-rutinoside, myo-inositol, quercetin, rutin, 2′-deoxy-D-ribose, sucrose, D-mannose and Alpha-D-glucose, were lowered (Table S3; Figure S1). In parallel, both ALA treatment and cold stress affected thirty-six pathways related to flavonoid, carbohydrate and amino acid metabolism (Figure 3C; Table S3).

Furthermore, we analyzed the content changes of all 67 metabolites associated with cold stress in tea leaves after ALA treatment (Figure 3B). The results show that ALA exerted the opposite effect on these metabolites compared with cold stress. The metabolites increased by cold stress were suppressed in tea leaves treated with ALA. In contrast, metabolites suppressed by cold stress were induced by ALA treatment (Figure 3B). These results imply that ALA might strengthen plant cold resistance by altering the levels of metabolites involved in plant cold resistance. Then, to evaluate the importance of these metabolites, we constructed a co-expression network between ALA-related and cold-related metabolites (Figure 3D). A detailed topological network was further visualized by Cytoscape to investigate the importance of metabolites in the network (Figure 3D). The topological network showed that the metabolites associated with ALA were closely correlated with cold-related metabolites. Catechin, 3,4-dihydroxyphenylacetic acid, procyanidin B2, betaine, cyanidin 3-galactoside cation, D-arabinono-1,4-lactone, myo-inositol and rutin are imperative in the network and termed hub metabolites (Figure 3D). Most of these metabolites are flavonoids or their derivatives, anthocyanins (Figure 3D; Tables S1 and S2).

### 2.4. ALA Strengthened Induced Cold Resistance through Altering Tea Leaves’ Metabolic Patterns

**Figure 4:**
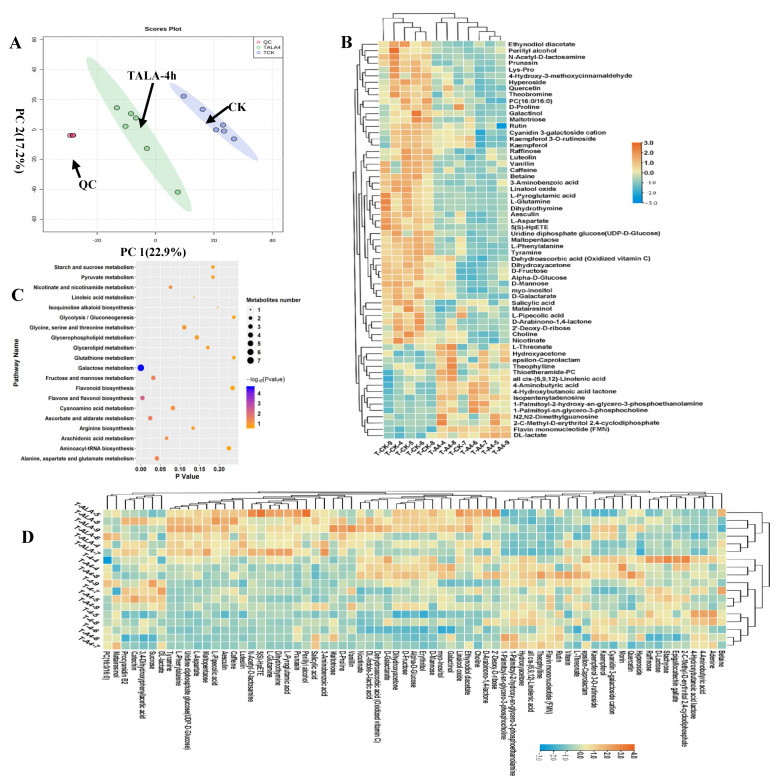
Metabolic alterations in ALA-treated tea tissue under cold stress. (**A**) PCA score plots for principal components 1 and 2 show high cohesion within groups and good separation among TALA-4h and CK groups. The sampling groups are color-coded as follows: green = TALA-4h; blue = CK; red = QC. (**B**) Heatmap visualization of relative levels of differential metabolites in the TALA-4h vs. CK comparison. The normalized value of peak areas of metabolites was used to represent the relative metabolite content and complete linkage hierarchical clustering. Each sample is visualized in a single column and each metabolite is represented by a single row. The up- and downregulated metabolites are shown in red and blue, respectively. (**C**) Enrichment of the differential metabolites from the TALA-4h vs. CK comparison with respect to distinct KEGG pathways. Differential metabolites were mapped to distinct metabolic pathways. Enrichment *p*-values were computed from a hypergeometric distribution. A *p*-value cutoff of 0.05 was selected as the threshold. The plot sizes represent the number of metabolites in each pathway term. (**D**) Relative levels of cold-related metabolites in ALA-treated tissues after cold stress challenge. The scale represents the normalized peak area values for each metabolite in the samples.

We analyzed the metabolic composition of ALA-treated tea leaves under cold stress (TALA-4h) using metabolomics; untreated tea leaves were used as the control (CK). PCA was deployed to assess the differences in metabolic profiles between TALA-4h and CK samples. In the two-dimensional PCA plot, we noticed that the QC samples were grouped together, suggesting a similar metabolic profile among QC samples. The entire analysis was stable and repeatable. The results showed that the samples from the TALA-4h and CK groups were separated into two distinct areas in the plot following their experimental treatments, indicating that each treatment sample had a relatively distinctive metabolic profile. Importantly, PCA plots clearly distinguished the TALA-4h samples from controls, explaining 40.1% variations of PC1 and PC2 between TALA-4h and CK (Figure 4A). This observation demonstrates that ALA-4h caused observable changes in the metabolic profiles of tea leaves. Additionally, compared with the T4h group, TALA-4h could be easily distinguished from T4h samples. The PCA plots represented by PC1 and PC2 explained 36% of the variation in metabolic compositions between groups. This observation suggests that ALA-treated tea leaves exhibited greater differences in metabolic profiles under cold stress than tea leaves without ALA treatment. In the TALA-4h vs. CK comparison, we identified sixty-three differential metabolites with VIP values > 1.0 from the PLSDA model (Figure 4C). The contents of rutin, PC (16:0/16:0), quercetin and various carbohydrates were altered in the TALA-4h group. Among these 63 differential metabolites, 48 were increased in the TALA-4h leaves, including dehydroascorbic acid, quercetin, α-D-glucose, PC (16:0/16:0), caffeine, D-galactarate, rutin, theobromine, tyramine and myo-inositol. In parallel, the upregulated metabolites included theophylline, 2-C-methyl-D-erythritol 2, 4-cyclodiphosphate, flavin mononucleotide (FMN), L-threonate, DL-lactate, cis-(6,9,12)-linolenic acid and thioetheramide-PC. Remarkably, all 63 metabolites associated with cold stress exhibited more dramatic changes in ALA-treated tissues under cold challenge. This result suggests that ALA plays a vital role in the induced resistance of plants in response to cold stress. Pathway enrichment analysis of these sixty-three metabolites showed that they are mainly involved in galactose metabolism, flavone and flavonol biosynthesis, ascorbate and aldarate metabolism, fructose and mannose metabolism, alanine, aspartate and glutamate metabolism, arachidonic acid metabolism, nicotinate and nicotinamide metabolism, cyanoamino acid metabolism, glycine, serine and threonine metabolism and arginine biosynthesis. Remarkably, galactose metabolism and flavone and flavonol biosynthesis were the top two significant pathways under the TALA-4h condition, suggesting their importance in ALA’s promotion of plant cold resistance.

In the TALA-4h vs. T4h comparison, we identified four downregulated metabolites: acetylcarnitine, maltotriose, malvidin 3-O-glucoside cation and raffinose, and eleven upregulated metabolites: 1-palmitoyl-sn-glycerol-3-phosphocholine, galactinol, myo-inositol, cyclohexylamine, luteolin, trans-3-coumaric acid, perillyl alcohol, FMN, DL-indole-3-lactic acid, resorcinol and pyruvaldehyde.

## 3. Discussion

Tea, made from tea leaves, is the second most popular beverage in the world. Tea leaves, flowers and seeds contain a variety of polyphenols, theanine, theine and vitamins [2,41]. However, the cultivation of tea plants is limited by various abiotic stresses, especially cold stress, which causes a series of metabolic changes in tea tissues. Therefore, elucidating the specific metabolic patterns of tea plants under cold stress contributes to the development of the tea industry, while finding an effective management strategy is critical for protecting tea from cold stress. ALA is known to promote plant growth and improve plant resistance under abiotic stress [33,34,35]. In this study, we found that cold stress caused dramatic alterations in the metabolic patterns of tea leaves, typically in compositions of galactose and decreases in flavonoid contents. We found that the exogenous application of ALA affected cold-related flavonoid and galactose metabolisms, leading to changes in the compositions of related metabolites. Most differential metabolites were shared among cold-treated and ALA-treated tea tissues. Importantly, we identified that ALA changed the accumulation of metabolites under cold stress, including catechin, 3,4-dihydroxyphenylacetic acid and procyanidin B2, and decreased contents of betaine, cyanidin 3-galactoside cation, D-arabinono-1,4-lactone, myo-inositol and rutin. In addition, soluble sugars such as galactose are also involved in the mechanisms of ALA-promoted cold resistance in tea.

Reactive oxygen species (ROS) are important signaling molecules that participate in multiple biological processes in plants. Thus, plants deployed ROS scavenging systems, represented by SOD, CAT and POD, to effectively prevent ROS accumulation in vivo [42]. Plant ROS scavenging systems are inhibited under cold stress, leading to the accumulation of intracellular ROS. ROS accumulation could aggravate the degree of membrane lipid peroxidation, destroying the integrity of membranes and affecting plant growth and development [43]. We identified that ALA effectively reduces the accumulation of ROS and MDA and alleviates the obstacle of chlorophyll synthesis in transforming uroporphyrin III (UROIII) to Proto IX (Proto IX) in plants under cold stress, which could strengthen plant resistance to cold stress. Moreover, cold stress significantly reduces the proteins involved in photosystem II (PSII) and photosystem I (PSI), leading to the degradation of chloroplasts in severe cases [44,45]. Chloroplast degradation reduces the activity of the APX enzyme, then suppresses the scavenging of H_2_O_2_ and causes changes in plant metabolism [43]. In this study, several chloroplast-related metabolisms were altered under cold stress, including flavonoid and carbohydrate metabolism, especially galactose biosynthesis. This result is consistent with previous research on various plants following cold challenge [15,18,19,20]. Flavonoids and their derived anthocyanins have antioxidant activities and scavenge ROS in vivo [16]. Various studies have demonstrated that flavonoids strengthen cold resistance in plants [17,18]. Thus, decreases may result in loss of cold resistance and ROS scavenging in tea plants. Of note, we found that ALA treatment also increased various flavonoid contents, especially catechin, 3,4-dihydroxyphenylacetic acid and procyanidin B2, supporting our hypothesis that ALA could enhance cold resistance in tea leaves through preventing decreases in related metabolites in plant tissues.

**Figure 5:**
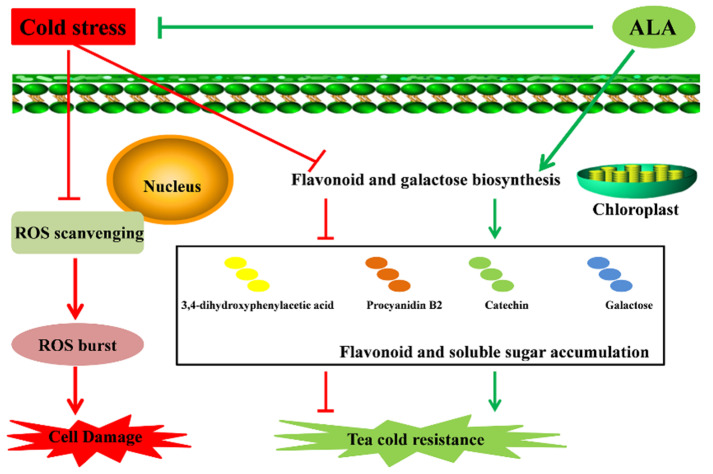
Possible pathways for cold stress tolerance in tea improved by ALA.

Moreover, ALA could improve cold resistance in plants by enhancing chlorophyll production, especially soluble sugar contents [46,47,48]. Contents of soluble sugar re important for plants in response to cold stress [19,20,21]. Various carbohydrates, especially soluble sugars, were shown to enhance plant cold resistance via increasing the concentration of cell fluid to reduce the freezing point of cytoplasm [25,26]. Here, we found that cold-treated tea plants exhibited changes in carbohydrates contents, particularly galactose-related metabolites. Soluble sugar could function as a signal to regulate plant cold resistance, and plants with cold resistance contain higher soluble sugar levels [19,20,27,28]. Interestingly, ALA treatment also rebuilds the composition of carbohydrates in tea plants, leading to opposite changes in the contents of cold-related compounds. Thus, we conclude that ALA deploys specific flavonoids, represented by catechin, 3,4-dihydroxyphenylacetic acid and procyanidin B2, and soluble carbohydrate galactose to enhance cold resistance in tea plants (Figure 5).

In the present study, we deciphered the detailed metabolic alterations in tea leaves under cold stress. We also examined the effect of exogenous ALA in promoting cold resistance through rebuilding metabolic compositions of tea plants. Cold stress mainly altered galactose metabolism and decreased flavonoid contents in tea tissues. Remarkably, ALA reversed metabolite changes associated with cold stress and primarily promoted cold resistance through increasing catechin, 3,4-dihydroxyphenylacetic acid and procyanidin B2 contents. Although our results did not identify cold-resistance genes for breeding cold-resistant tea plants, we fully demonstrated that ALA could be applied to improve cold resistance in tea plants. Overall, the present study provides a novel and valuable strategy for protecting tea plants from cold stress through the application of ALA.

## 4. Materials and Methods

### 4.1. Sample Preparation and Collection

The tea plant cultivar *Camellia sinensis* var. *Shaancha No.1* was used in this study. Tea plant seedlings were provided by the Tea Laboratory of Hanzhong Institute of Agricultural Sciences, Shaanxi Province (longitude: 107°2′12″ E, latitude: 32°27′36″ N). Two-year-old tea plant seedlings with consistent growth and size were selected and planted in plastic pots. There were three biological replicates in each pot and each treatment contained nine pots. Except for the control (CK, normal growth), there were three treatments: 5-ALA (30 mg/L) pretreatment for two days (TALA) and cold stress (−4 °C, T4h) and 5-ALA (30 mg/L) pretreatment for two days with cold stress (TALA-4h). For the chemical preparation, a solution of 5-ALA (Sigma, St Louis, MO, USA) was prepared using water. Then, 5-ALA (30 mg/L) was sprayed on tea plant seedling leaves until they were wet. The CK treatments were treated with water. Before cold treatment, all plants were planted in an artificial climate box (25 °C, 12 h light/12 h dark). For the cold treatment, tea plant seedlings were transferred from 25 °C to −4 °C. Twelve hours after −4 °C treatment, the leaves were harvested from all seedlings, and then all samples were quickly frozen with liquid nitrogen before storing at −80 °C.

### 4.2. Metabolite Extraction

Each experimental group contained six biological replicates to ensure the validity of cold-induced metabolic variations. First, 100 mg of the ground sample was transferred to an Eppendorf tube. After adding 400 μL of extract solution (acetonitrile: methanol = 1: 1, containing isotopically labeled internal standard mixture), the samples were vortexed for 30 s, sonicated for 10 min in an ice-water bath, and incubated at −40 °C for one hour to precipitate proteins. Then the sample was centrifuged at 12000 rpm (Centrifuge 5418R, Eppendorf, Darmstadt, Germany) at 4 °C for 15 min. The resulting supernatant was transferred to a fresh glass vial for analysis. Finally, the quality control (QC) sample was prepared by mixing an equal aliquot of the supernatants from all samples [49].

### 4.3. LC–MS/MS Analysis

LC–MS/MS analyses were performed using a UHPLC system (Vanquish, Thermo Fisher Scientific) with a UPLC BEH Amide column (2.1 mm × 100 mm, 1.7 μm) coupled to a Q Exactive HFX mass spectrometer (Orbitrap MS, Thermo, Waltham, MA USA). The mobile phase consisted of 25 mmol/L ammonium acetate and 25 mmol/L ammonia hydroxide in water (pH = 9.75) (A) and acetonitrile (B). The auto-sampler temperature was 4 °C and the injection volume was 3 μL. The QE HFX mass spectrometer was used for its ability to acquire MS/MS spectra in the information-dependent acquisition (IDA) mode in the control of the acquisition software (Xcalibur, Thermo, Waltham, MA, USA). The acquisition software continuously evaluated the full scan MS spectrum in this mode. The ESI source conditions were set as follows: sheath gas flow rate as 30 Arb, Aux gas flow rate as 25 Arb, capillary temperature 350 °C, full MS resolution as 60,000, MS/MS resolution as 7500, collision energy as 10/30/60 in NCE mode, spray Voltage as 3.6 kV (positive) or −3.2 kV (negative), respectively [50].

### 4.4. Data Preprocessing and Annotation

The raw data were converted to the mzXML format using ProteoWizard and processed with an in-house program developed using R and based on XCMS for peak detection, extraction, alignment and integration. The in-house database was established with available authentic standards. Then, an in-house MS2 database (BiotreeDB) was applied in metabolite annotation. The cutoff for annotation was set at 0.3. The data variation was performed by R packages XCMS software [51]. The Principal Component Analysis (PCA) and Partial Least Squares Discriminant Analysis (PLSDA) were performed using Metaboanalyst 3.0. PCA and PLSDA were performed on the normalized peak area of the metabolites in each sample. The annotated data were treated by mean centering and unit variance scaling. Therapy *p* < 0.05 and fold change > 2.0 were used to identify significantly differential metabolites. The VIP values for metabolites were calculated using PLSDA analysis.

### 4.5. Pathway Enrichment Analysis and Co-Expression Network Construction

After testing the enrichment of the differentially expressed metabolites using a two-tailed Fisher’s exact test, the Kyoto Encyclopedia of Genes and Genomes (KEGG) database was used to identify enriched pathways. The pathway analysis was performed using Metaboanalyst 3.0. A pathway with a *p*-value < 0.05 was considered significant. The correlations among all differentially expressed metabolites associated with cold stress and ALA treatments were analyzed using an in-house R script based on the Spearman method [42]. A *p*-value < 0.05 and a correlation value > 0.7 were set as the thresholds to identify hub relationships in the metabolite network. The topological network was further constructed and visualized using Cytoscape 3.0.

## 5. Conclusions

Cold stress could induce changes in the compositions of soluble sugars and flavonoids in tea tissues, then block the ROS scavenging ability and inhibit the growth of tea plants. Remarkably, exogenous application of 5-aminolevulinic acid (ALA) effectively strengthened cold resistance in tea plants. ALA treatment increased the contents of flavonoids associated with cold stress in tea tissues, especially catechin, 3,4-dihydroxyphenylacetic acid and procyanidin B2. In addition, the soluble sugar galactose was also found to perform a function in the mechanisms of ALA-promoted cold resistance in tea plants. In the future, the regulatory mechanisms associated with the accumulation of the above metabolites induced by exogenous ALA require further elucidation. This work will support efforts to enhance cold tolerance in tea plants.

## Data Availability

The data presented in this study are available on request from the corresponding author. The data are not publicly available due to all data generated or analysed during this study are included in this published article and its supplementary information files.

## Supplementary Materials

The following supporting information can be downloaded at: https://www.mdpi.com/article/10.3390/metabo12050392/s1, Figure S1: Heatmap displaying the relative levels of metabolites shared in T4h vs. CK and TALA vs. CK comparisons. The upregulated and downregulated metabolites are shown in red and blue, respectively, Figure S2: PCA plots of metabolic profiles from the TALA-4h and T4h groups. The samples from TALA-4h and T4h are represented by green and red plots, respectively. The ellipse is the 95% confidence interval, Figure S3: OPLS-DA analysis of each pairwise comparison (CK vs. T4h and TALA vs. TALA4). OPLS-DA score plots significantly identified the differences in each pairwise comparison. All samples were within 95% confidence intervals (Hotelling’s T-squared ellipse), Table S1: Upregulated and downregulated differential metabolites between CK and after −4 °C treatment, Table S2: Differential metabolites between CK and after ALA treatment, Table S3: Differential metabolites after ALA treatments or cold stress alone.
