# Supplementary material for: Metabolomics Reveals 5-Aminolevulinic Acid Improved the Ability of Tea Leaves (Camellia sinensis L.) against Cold Stress
Source: Metabolites. 2022 Apr 26;12(5):392. doi: 10.3390/metabo12050392 (PMC9144897; doi:10.3390/metabo12050392)
Supplement: Supplementary file 1 [file metabolites-12-00392-s001.zip › Figure S1.pdf]

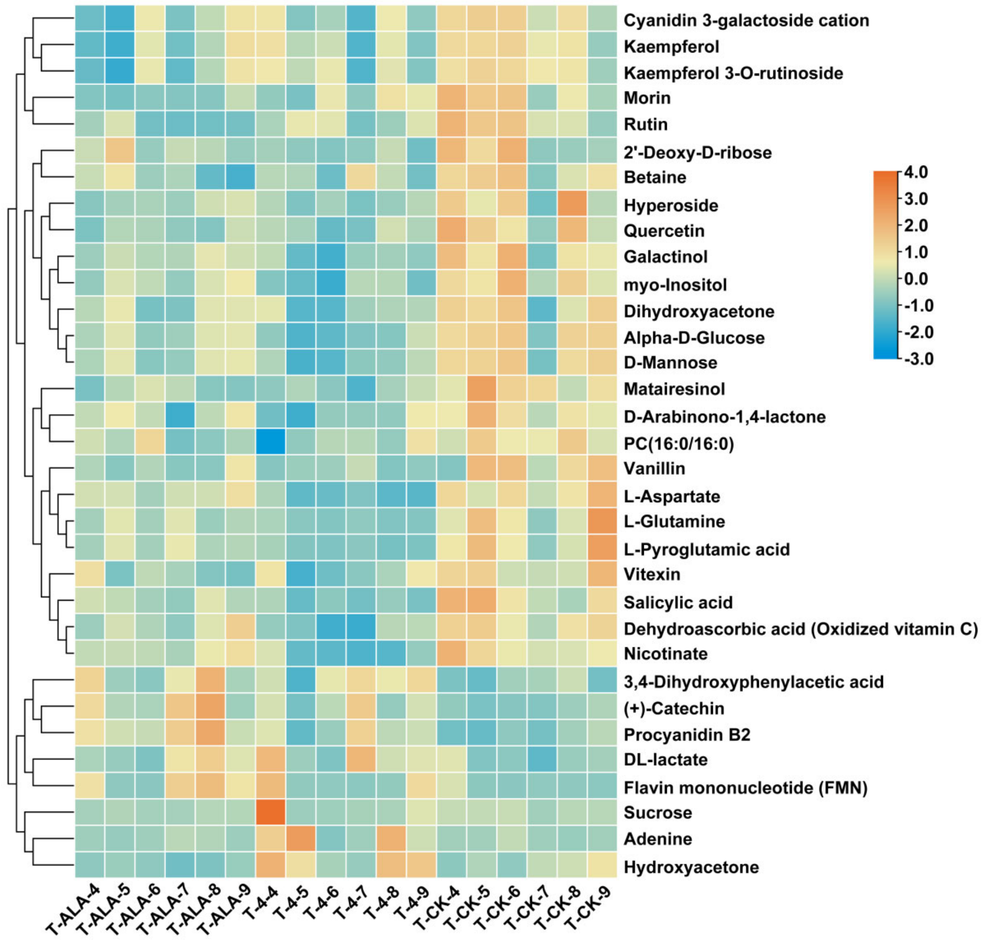

Figure S1. Heatmap displaying the relative levels of metabolites shared in T4h vs. CK and TALA vs. CK comparisons. The upregulated and downregulated metabolites are shown in red and blue, respectively.
