# Supplementary material for: Metabolomics Reveals 5-Aminolevulinic Acid Improved the Ability of Tea Leaves (Camellia sinensis L.) against Cold Stress
Source: Metabolites. 2022 Apr 26;12(5):392. doi: 10.3390/metabo12050392 (PMC9144897; doi:10.3390/metabo12050392)
Supplement: Supplementary file 1 [file metabolites-12-00392-s001.zip › Figure S2.pdf]

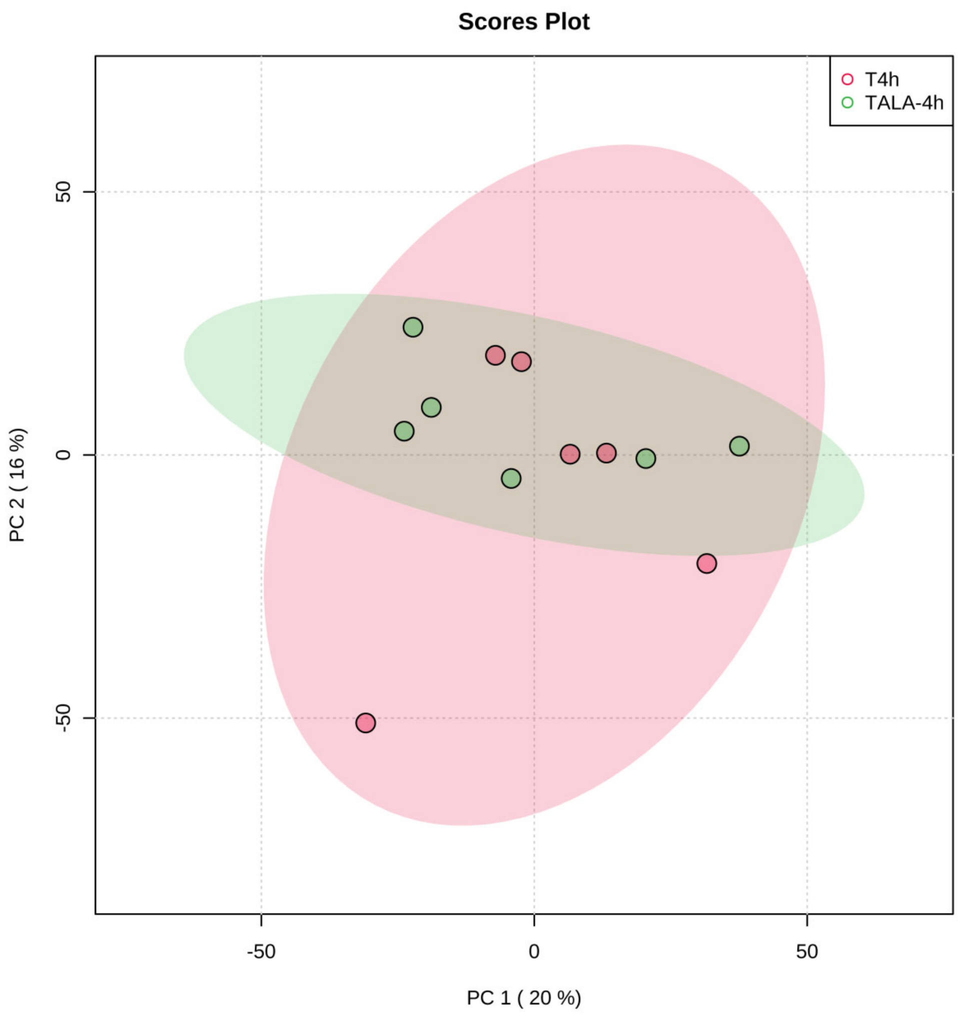

Figure S2. PCA plots of metabolic profiles from the TALA-4h and T4h groups. The samples from TALA-4h and T4h are represented by green and red plots, respectively. The ellipse is the 95% confidence interval.
