# Supplementary material for: Metabolomics Reveals 5-Aminolevulinic Acid Improved the Ability of Tea Leaves (Camellia sinensis L.) against Cold Stress
Source: Metabolites. 2022 Apr 26;12(5):392. doi: 10.3390/metabo12050392 (PMC9144897; doi:10.3390/metabo12050392)
Supplement: Supplementary file 1 [file metabolites-12-00392-s001.zip › Figure S3.pdf]

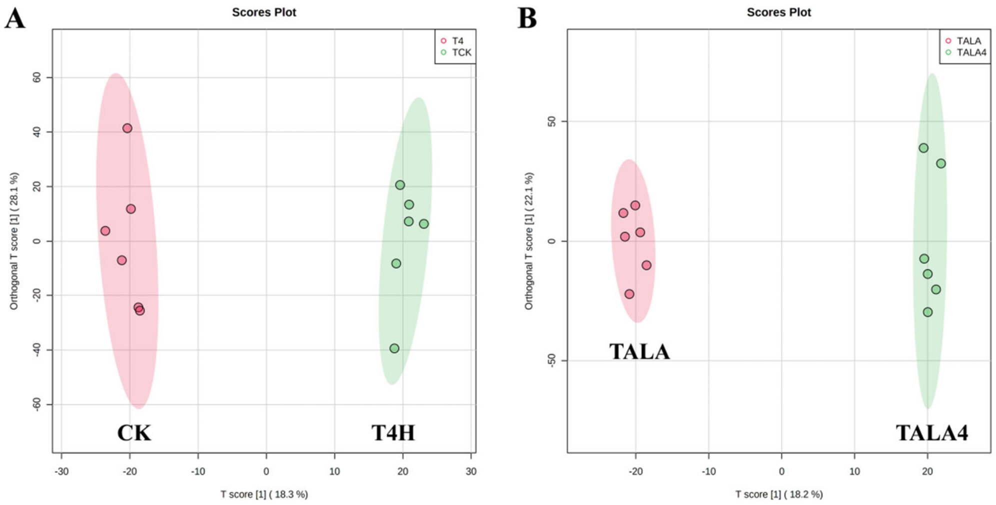

Figure S3. OPLS-DA analysis of each pairwise comparison (CK vs. T4H and TALA vs. TALA4). OPLS-DA score plots significantly identified the differences in each pairwise comparison. All samples were within 95% confidence intervals (Hotelling's T-squared ellipse).
